# Multiple Interferon Stimulated Genes Synergize with the Zinc Finger Antiviral Protein to Mediate Anti-Alphavirus Activity 

**DOI:** 10.1371/journal.pone.0037398

**Published:** 2012-05-16

**Authors:** Sophiya Karki, Melody M. H. Li, John W. Schoggins, Suyan Tian, Charles M. Rice, Margaret R. MacDonald

**Affiliations:** 1 Laboratory of Virology and Infectious Disease, The Rockefeller University, New York, New York, United States of America; 2 Center for Clinical and Translational Science, The Rockefeller University, New York, New York, United States of America; Utah State University, United States of America

## Abstract

The zinc finger antiviral protein (ZAP) is a host factor that mediates inhibition of viruses in the *Filoviridae*, *Retroviridae* and *Togaviridae* families. We previously demonstrated that ZAP blocks replication of Sindbis virus (SINV), the prototype *Alphavirus* in the *Togaviridae* family at an early step prior to translation of the incoming genome and that synergy between ZAP and one or more interferon stimulated genes (ISGs) resulted in maximal inhibitory activity. The present study aimed to identify those ISGs that synergize with ZAP to mediate *Alphavirus* inhibition. Using a library of lentiviruses individually expressing more than 350 ISGs, we screened for inhibitory activity in interferon defective cells with or without ZAP overexpression. Confirmatory tests of the 23 ISGs demonstrating the largest infection reduction in combination with ZAP revealed that 16 were synergistic. Confirmatory tests of all potentially synergistic ISGs revealed 15 additional ISGs with a statistically significant synergistic effect in combination with ZAP. These 31 ISGs are candidates for further mechanistic studies. The number and diversity of the identified ZAP-synergistic ISGs lead us to speculate that ZAP may play an important role in priming the cell for optimal ISG function.

## Introduction

Viruses in the *Alphavirus* genus (*Togaviridae* family) are arthropod-borne viruses, which can infect a variety of birds and mammals [1]. Humans, when inoculated through the bite of an infected arthropod, support virus replication and can develop severe disease including fever, debilitating arthritis, encephalitis, and death. There are currently no licensed vaccines or specific therapies available for prevention or treatment of diseases caused by these important pathogens. The zinc finger antiviral protein (ZAP, gene symbol ZC3HAV1), a host protein originally identified in a screen as inhibitory to the retrovirus Moloney murine leukemia virus [2], is able to inhibit the replication of multiple *Alphavirus* genus members [3]–[6] when overexpressed in cultured cells. ZAP also inhibits members of the *Filoviridae* family [7], but is not able to inhibit all viruses [3]. ZAP mediates its antiviral activity by binding to viral RNA [8], [9], which for MMLV results in exosome-mediated degradation of the viral RNA [10] in a cellular process involving host helicases [11], [12]. Using Sindbis virus (SINV), the well-studied prototype alphavirus, we demonstrated that ZAP blocks an early step after entry and prior to production of the viral polyprotein [3] and that the inhibitory activity requires ZAP self association [13]. ZAP is expressed via alternative splicing as two distinct isoforms, with the longer of the two showing greater anti-alphaviral activity [4].

The interferon (IFN) proteins are generated and secreted in response to triggering of sensors within the cell that recognize pathogen-associated molecular patterns (PAMPs), including viral nucleic acid [14]–[16]. PAMPs bind to pattern recognition receptors, consisting of cell surface or endosomally located Toll-like receptors and cytosolic sensors. Upon binding, activation results in a cascade of signaling events resulting in the phosphorylation, activation, and nuclear translocation of the transcription factors nuclear factor kappa-light-chain-enhancer of activated B cells (NF-κB), interferon regulatory factor (IRF)-3 and in some cell types, IRF-7. Transcriptional upregulation of the IFN-β gene by IRF-3 and NF-κB results in IFN-β production, which, upon secretion, binds to and signals through specific Type I IFN cell surface receptors through both autocrine and paracrine mechanisms. Phosphorylation, heterodimerization and nuclear translocation of the latent signal transducers and activators of transcription (STAT)1 and STAT2 transcription factors results in the upregulation of hundreds of IFN-stimulated genes (ISGs), which confer upon the cell an antiviral state [17]. A family of IFN-α genes, which also signal through the Type I IFN receptor, is induced in cells expressing IRF-7, which itself is induced by Type I IFN signaling, allowing for signal amplification at later stages following infection or for early production of large amounts of IFN-α in cell types that constitutively express IRF-7. The IFN-λs [18], [19] are another class of IFNs that mediate STAT1/STAT2-dependent signaling after binding to the IFN-λ receptor, resulting in a similar upregulation of antiviral genes.

ZAP is induced by treatment of cells with interferon (IFN)-α/β [5], [20] or IFN-λ [20], and its expression is also upregulated upon viral infection [5], [21]. Moreover, silencing of ZAP during IFN-α/β treatment diminishes IFN's ability to establish a cellular antiviral state against SINV [6], [22]. Interestingly, ZAP expression is also directly induced by IRF-3 activation, and the short isoform interacts with the intracellular pattern recognition receptor retinoic acid-inducible gene-I (RIG-I) to enhance IFN-β production. Thus ZAP is a key component of the host cell's response to viral infection potentially working at multiple levels to confer resistance to alphavirus infection. Evidence suggests that ZAP works in concert with other ISGs to confer maximal protection against alphavirus infection. Previously, using gene silencing approaches, it was shown in murine embryonic fibroblasts (MEFs) that ZAP and ISG20 together provided greater control of SINV replication than either ZAP or ISG20 alone [6]. In BHK-21 hamster fibroblasts, which are likely defective in IFN production [23]–[27], we found that ZAP overexpression failed to block SINV virion production, despite having a 10-fold effect on viral polyprotein expression, and failed to prevent SINV-mediated cell death [22]. Pretreatment with IFN-α, however, restored ZAP's antiviral and protective activity in a dose-dependent manner. In that work, we demonstrated that expression of the amino terminal domain of ZAP (NZAP) in BHK-21 cells failed to reduce viral titers and pretreatment with IFN (100 U/ml) only reduced titers by ∼1 log. However, expression of NZAP and pretreatment with IFN together resulted in a synergistic inhibition of virion production, with an ∼3 log reduction after high moi infection (moi = 5) and >4 log reduction upon low moi infection (moi = 0.01). Synergistic activity was also noted in MEFs, where IFN treatment or NZAP expression each reduced virion production by ∼2 logs, while together a >4 log reduction was noted. Thus one or more ISGs are able to work in concert with ZAP for maximal virus inhibition.

In this study, we set out to identify which ISGs synergize with ZAP to confer an antiviral state effective against infection with SINV. Using a library of lentiviruses individually expressing 383 ISGs [28], we screened for ISG-mediated anti-SINV activity in BHK-21 cells in the presence or absence of overexpressed rat NZAP. Our results demonstrate that 69 of the tested ISGs demonstrated a synergistic antiviral activity with ZAP. Follow-up studies on the ISGs that blocked SINV in either cell type and/or were potentially synergistic with ZAP in the initial screen verified that 31 demonstrated a statistically significant synergy with ZAP. The information will be utilized for future mechanistic studies aimed at developing novel treatment or preventative strategies for these important human pathogens in the *Alphavirus* genus.

## Results

### Differential ISG antiviral effect in BHK-21 cells with or without ZAP overexpression

We utilized our previously described [22] IFN defective BHK-21 cell derivatives expressing the Zeocin resistance gene (BHK/HA-Zeo, control cells) or the active amino terminus of rat ZAP fused to the zeocin resistance gene (BHK/NZAP-Zeo, ZAP cells) to evaluate the efficacy of individual ISGs against SINV. Using lentiviruses to individually express a library of ISGs [28], we tested for inhibition of SINV replication in the absence (BHK/HA-Zeo) or presence (BHK/NZAP-Zeo) of overexpressed NZAP. The library was constructed such that a bicistronic message expressed the ISG of interest followed by the red fluorescent protein TagRFP under the control of an internal ribosome entry sequence (IRES). For each ISG, one well of each cell type was transduced with the appropriate VSV-G pseudotyped lentiviral particles, and 2 d later the cells were challenged with SINV (TE/5′2J/GFP) expressing enhanced green fluorescent protein (EGFP). We utilized a lentivirus expressing Firefly luciferase (Fluc) as a negative control not expected to affect SINV replication. After 8 h of infection, cells were harvested and analyzed by flow cytometry to determine the number of GFP positive (GFP+) cells within the transduced (TagRFP+) population.

**Figure 1:**
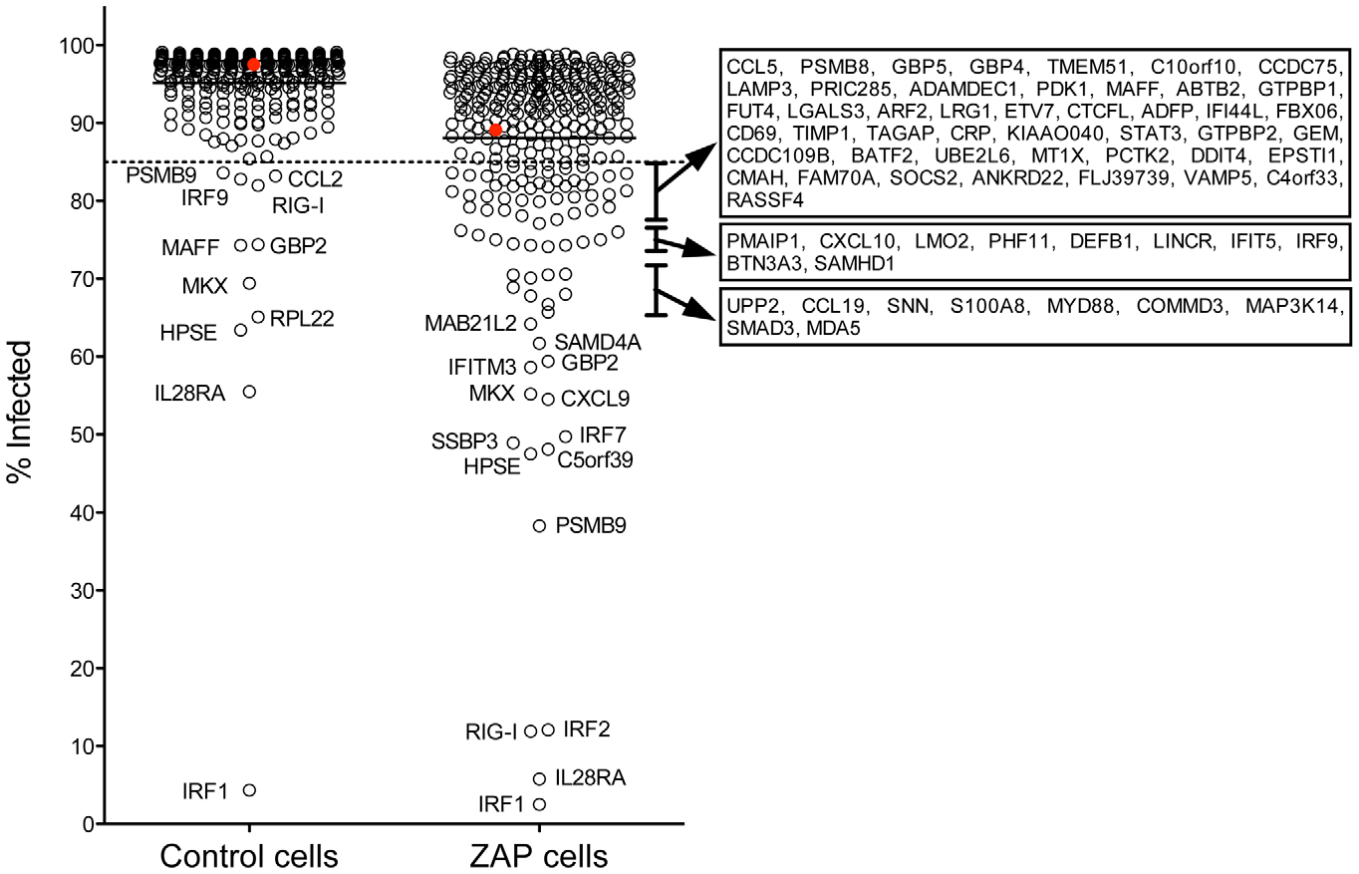
Anti-SINV activity of a library of 383 ISGs in control and ZAP-expressing cells. BHK/HA-Zeo (Control) cells or BHK/NZAP-Zeo cells expressing the amino terminal domain of rat ZAP (ZAP cells) were transduced with lentiviruses co-expressing individual ISGs and the red fluorescent protein TagRFP. After 2 d, the cells were challenged with SINV expressing GFP (moi = 5). After 8 h, the cells were harvested and analyzed by flow cytometry to determine the percentage of infected cells (GFP+) within the transduced (RFP+) population. Red symbols indicate cells expressing the control protein, Fluc, while black open circles indicate cells expressing the individual ISGs. For each cell type, the line in the scatter plot indicates the mean value for the percentage of infected cells. Gene symbols are shown for ISGs resulting in infection rates below an arbitrary cutoff of 85% (dashed line).

Of the 383 ISGs tested, 308 and 311 met our criteria for analysis (≥5,000 cells analyzed, ≥30% transduced) in the control, and ZAP cells, respectively. In control cells expressing Fluc, 97.5% of the cells were infected, while 89.1% of the ZAP cells expressing Fluc were infected. Amongst all the ISGs, the mean infection percentage in the control cells was 95.2 (SD = 7.4). Expression of 11 ISGs resulted in infection levels of less than an arbitrary cutoff of 85% (Fig. 1). ISGs that demonstrated the most potent SINV inhibition in the control cells were IRF1, IL28RA, HPSE, RPL22, MKX, MAFF, GBP2, RIG-I (also known as DDX58), IRF9, CCL2, and PSMB9. These ISGs constitute anti-SINV ISGs that can function independently of ZAP overexpression. In contrast, in the ZAP cells, where the mean infection percentage was 88.1 (SD = 13.5), expression of 80 ISGs, which constitutes more than one quarter of those ISGs analyzed, resulted in infection levels of less than 85% (Fig. 1). Thus expression of ZAP in concert with other individual ISGs effectively promoted the antiviral state of these cells. It should be noted that ISGs with antiviral activity in control cells might not appear as antiviral in ZAP cells due to infection rates being near the 85% cutoff (e.g. CCL2), or due to a failure to meet our analysis criteria in ZAP cells (e.g. RPL22). The percentage of infected cells for each ISG in the two cell types is provided in Supplementary Table S1.

### Comparison of ISG antiviral activity in control cells versus ZAP-expressing cells and assessment for synergy

**Figure 2 pone-0037398-g002:**
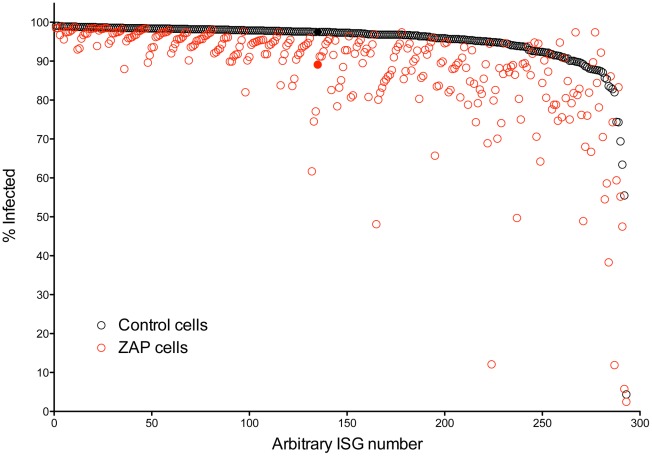
Comparison of the antiviral activity of 292 ISGs in the control cells versus ZAP-expressing cells. For those 292 ISGs with infection data in each cell type the results were sorted based on the percentage of infected cells in the control cells and the paired results are plotted versus an arbitrary ISG number. For each ISG, black circles show the % infected in the control cells while red circles show the % infected in the ZAP cells. Results obtained in the absence of ISG expression (Fluc) are shown with the filled circles.

Of the 383 ISGs contained in the library, a total of 292 met our criteria for analysis (≥5,000 cells analyzed, ≥30% transduced) in both control and ZAP cells. Fig. 2 shows the paired results for each ISG in the two cell types, plotted in order by the percentage of cells infected in the control cells. As noted above, expression of ZAP without an ISG (Fluc control) resulted in a decrease from 97.5% infected to 89.1% infected (an 8.4% reduction, black and red solid symbols, respectively). Compared to the inhibitory results seen in the control cells, a large number of ISGs had an apparently greater inhibitory effect in the ZAP-expressing cells, as can be seen by the many data points (red open circles) falling well below the corresponding result in the control cells.

To explore whether a given ISG and ZAP exhibited synergistic antiviral activity, we utilized a Poisson regression model. The observed rate of infection for a given ISG was calculated from the four quadrant (RFP versus GFP) flow cytometric plots as the number of double positive cells (Quadrant 2) divided by the total number of RFP+ cells (Quadrants 1+2), and was assessed for a statistically significant negative coefficient of the interaction term of ZAP and ISG in the model (see Methods). A total of 69 ISGs were found to exhibit statistically significant antiviral synergy in combination with ZAP (adjusted P<0.05, Table 1). The complete list of the synergy testing results on the 292 ISGs is available in Supplementary Table S1.

**Table 1:** Significant estimated interaction coefficients from Poisson regression of the primary screen results and the corresponding P values.

Gene Symbol	Interaction Coefficient	P value	Adjusted P value^1^
**IL28RA** 2	−2.4228	0	0
**RIG-I**	−2.1559	0	0
**IRF2**	−2.1088	0	0
**IRF1**	−0.8664	0	0
**PSMB9**	−0.733	0	0
**C5orf39**	−0.6977	0	0
**IRF7**	−0.6034	0	0
**SSBP3**	−0.5504	0	0
**SAMD4A**	−0.4467	0	0
**CXCL9**	−0.4015	0	0
**MDA5**	−0.3381	0	0
**IFITM3**	−0.3163	0	0
**MAB21L2**	−0.3131	0	0
**MYD88**	−0.2517	0	0
HPSE	−0.2507	0	0
**COMMD3**	−0.2358	0	0
**S100A8**	−0.2357	0	0
**UPP2**	−0.2207	0	0
**SMAD3**	−0.2169	0	0
GBP2	−0.2151	0	0
MKX	−0.2128	0	0
**IFIT5**	−0.201	0	0
**SAMHD1**	−0.1927	0	0
**BTN3A3**	−0.1764	0	0
**RASSF4**	−0.1761	0	0
SNN	−0.1592	0	0
**PHF11**	−0.1537	0	0
**FLJ39739**	−0.1469	0	0
KIAA0040	−0.1324	0	0
IFI44L	−0.123	0	0
STAT3	−0.1212	0	0
TAGAP	−0.1212	0	0
LINCR	−0.119	0	0
PCTK2	−0.1172	0	0
GTPBP2	−0.1147	0	0
FAM70A	−0.1126	0	0
DEFB1	−0.1118	0	0
EPSTI1	−0.1116	0	0
LMO2	−0.1108	0	0
GEM	−0.1033	0	0
C4orf33	−0.0992	0	0
GTPBP1	−0.0979	0	0
DDIT4	−0.0951	0	0
ANKRD22	−0.0943	0	0
FBXO6	−0.0939	0	0
MT1X	−0.0925	0	0
PMAIP1	−0.0906	1.00E−04	1.00E−04
ETV7	−0.09	1.00E−04	2.00E−04
ABTB2	−0.0885	1.00E−04	3.00E−04
SOCS2	−0.085	1.00E−04	1.00E−04
ADFP	−0.0818	3.00E−04	6.00E−04
UBE2L6	−0.0762	3.00E−04	6.00E−04
LAMP3	−0.0747	2.00E−04	4.00E−04
ARG2	−0.0734	3.00E−04	6.00E−04
LRG1	−0.07	3.00E−04	6.00E−04
VAMP5	−0.066	0.0037	0.0058
CCDC109B	−0.0657	0.0016	0.0028
CXCL10	−0.0656	0.0023	0.0037
GCH1	−0.0642	9.00E−04	0.0016
GBP5	−0.0629	0.0032	0.0052
PRIC285	−0.0613	0.003	0.0049
ADAMDEC1	−0.0583	0.0046	0.0071
PDK1	−0.0582	0.0092	0.0135
CMAH	−0.0531	0.0069	0.0102
ANGPTL1	−0.0477	0.0232	0.0322
CCDC75	−0.0455	0.0175	0.0247
ERLIN1	−0.0451	0.0271	0.0374
DHX58	−0.0449	0.0323	0.0436
BATF2	−0.0448	0.0303	0.0415

1 The P values were adjusted using the BH procedure to adjust for multiple comparisons.

2 ISGs listed in bold demonstrated the largest reduction in percentage of infected cells upon coexpression of ZAP and were chosen for the initial follow up confirmatory testing.

### Confirmation of the synergistic hits

**Figure 3 pone-0037398-g003:**
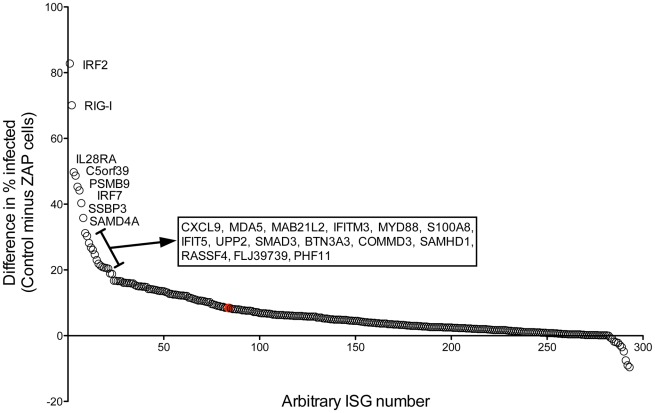
Reduction in the percentage of infected cells by ZAP. For each ISG the reduction in the percentage of infected cells due to ZAP co-expression was calculated by subtracting the percentage of infected cells in the ZAP cells from the percentage infected in the control cells. After sorting, the differences were plotted versus an arbitrary ISG number. The difference seen between the control and ZAP cells in the absence of ISG expression (Fluc) is shown by the red symbol. Gene symbols are shown for the 23 ISGs with the greatest difference in infection percentage (≥18) due to ZAP expression.

To confirm the synergistic activity of ISGs with ZAP against SINV infection, we took two approaches to select genes for follow-up screening. First, we focused on the difference in the percentage of infected cells in the control cells compared to the ZAP cells (% infected in control minus % infected in ZAP). Fig. 3 shows the calculated differences, with the ISGs ranked according to the size of the difference. Because our primary screen was performed with just a single well for each ISG in the two cell types, we chose those with the largest difference to confirm the potential synergistic effect. Of the 292 ISGs interrogated in both cell types, the top 23, all of which showed statistically significant synergy in the primary screen (Table 1), were chosen for validation.

**Figure 4:**
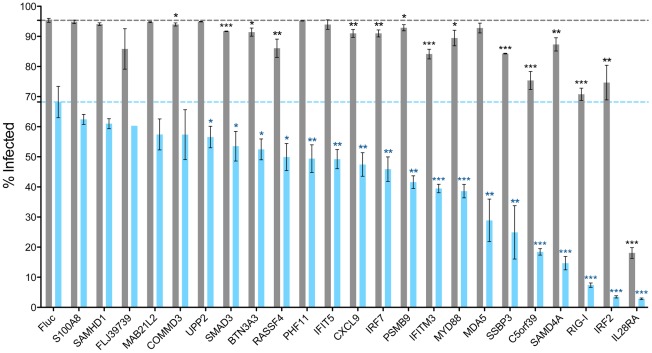
Confirmatory testing of the top ISG hits synergizing with ZAP. Triplicate wells of BHK/HA-Zeo cells (Control cells, gray bars) or BHK/NZAP-Zeo cells expressing the amino terminal domain of rat ZAP (ZAP cells, blue bars) were transduced with lentiviruses co-expressing the indicated ISGs and the red fluorescent protein TagRFP. After 2 d, the cells were challenged with SINV expressing GFP (moi = 5). After 8 h, the cells were harvested and analyzed by flow cytometry to determine the percentage of infected cells (GFP+) within the transduced (RFP+) population. Mean values are plotted; error bars indicate the standard deviation. Dashed lines indicate the percentage of infection determined in control cells expressing Fluc (gray) or ZAP cells expressing Fluc (blue). For FLJ39739 transduction of ZAP cells, there was only one replicate for analysis. Asterisks indicate mean values statistically different than values obtained in Fluc-expressing cells for the corresponding cell type (unpaired *t* test, *, P<0.05; **, P<0.01; ***, P<0.001).

For each ISG chosen for confirmatory testing (IRF2, RIG-I, IL28RA, C5orf39, PSMB9, IRF7, SSBP3, SAMD4A, CXCL9, MDA5 (also known as IFIH1), MAB21L2, IFITM3, MYD88, S100A8, IFIT5, UPP2, SMAD3, BTN3A3, COMMD3, SAMHD1, RASSF4, FLJ39739, PHF11) we prepared newly packaged VSV-G pseudotyped lentiviral particles, transduced the control and ZAP cells, and 2 days later challenged the cells with GFP-expressing SINV. As can be seen in Fig. 4, 98.3% of the control cells expressing Fluc were infected, while 68.2% of the ZAP cells expressing Fluc were infected. Thus ZAP expression reduced the percentage of infected cells by 27.1 (compared to 8.4 in the primary screen). In the absence of ZAP (control cells, gray bars) none of the tested ISGs reduced the percentage of infected cells to levels below that seen upon ZAP expression (ZAP cells expressing Fluc) with the exception of IL28RA, which reduced infection to 18.1% of the cells. However, there were significant reductions in the percentages of control cells infected upon expression of 15 of the 23 ISGs including COMMD3, BTN3A3, PSMB9, MYD88 (P<0.05), RASSF4, CXCL9, IRF7, SAMD4A, IRF2 (P<0.01), SMAD3, IFITM3, SSBP3, C5orf39, RIG-I, and IL28RA (P<0.001). Similar to our findings in the primary screen (Figs. 2 and 3), for each of the ISGs tested, antiviral activity was enhanced in the ZAP cells (Fig. 4, blue bars), suggesting synergistic or additive activity against SINV between ZAP and the individual ISGs. Of the 23 ISGs tested, significant reductions in the percentages of ZAP cells infected were obtained upon expression of 18 ISGs, which included UPP2, SMAD3, BTN3A3, RASSF4 (P<0.05), PHF11, IFIT5, CXCL9, IRF7, PSMB9, MDA5, SSBP3 (P<0.01), IFITM3, MYD88, C5orf39, SAMD4A, RIG-I, IRF2 and IL28RA (P<0.001). Amongst the tested ISGs, reduction in the percentage of infected cells by ZAP co-expression (% infected in the control cells minus % infected in the ZAP cells) ranged from 15.2 (IL28RA) to 72.7 (SAMD4A) with an average reduction of 45.4 (±14.3). The values for the replicates of the confirmatory test are available in Supplementary Table S2.

To ascertain whether the ISGs displayed synergistic activity with ZAP against SINV, we utilized a two way ANOVA model to estimate the ZAP×ISG interaction coefficient and corresponding P value for each ISG (see Methods and Supplementary Table S2). After adjustment of the P values for multiple comparisons (Hommel's adjustment) 16 of the 23 ISGs were found to exhibit statistically significant antiviral synergy in combination with ZAP (negative interaction coefficient and adjusted P<0.05, Table 2). Thus IRF2, RIG-I, C5orf39, PSMB9, IRF7, SSBP3, SAMD4A, CXCL9, MDA5, IFITM3, MYD88, IFIT5, UPP2, SMAD3, BTN3A3, and PHF11 can each mediate a synergistic effect in combination with ZAP to confer an anti-alphavirus state upon the cell. Although a statistically significant synergistic effect was not detected for ZAP and IL28RA, a potent antiviral state was obtained upon coexpression that was greater than that seen for either of the factors expressed individually.

**Table 2:** Estimated interaction coefficients from ANOVA of the 23 selected ISGs and corresponding P values.

Gene Symbol	Estimate (%)	P value	Adjusted P value^1^
**SAMD4A** 2	−45.5333	0	0
**IRF2**	−44.0567	0	0
**MDA5**	−36.7333	0	0
**RIG-I**	−36.2433	0	0
**SSBP3**	−32.2667	0	0
**C5orf39**	−29.8	0	0
**PSMB9**	−24.1333	0	0
**MYD88**	−23.7333	0	0
**PHF11**	−18.6333	0	1.00E−04
**IRF7**	−17.9667	0	2.00E−04
**IFIT5**	−17.5667	0	2.00E−04
**IFITM3**	−17.5333	0	2.00E−04
**CXCL9**	−16.4	1.00E−04	7.00E−04
**BTN3A3**	−11.8333	0.0031	0.023
**UPP2**	−11.2	0.0051	0.0355
**SMAD3**	−11.0333	0.0057	0.0374
MAB21L2	−10.1667	0.0107	0.0534
COMMD3	−9.4667	0.0172	0.0688
RASSF4	−9	0.0233	0.0932
SAMHD1	−5.9333	0.1318	0.2707
S100A8	−5.2667	0.1804	0.3609
FLJ3973	1.6	0.7386	0.7386
IL28RA	11.92	0.0029	0.023

1 The P values were adjusted using Hommel's adjustment for multiple comparisons.

2 Genes in bold font showed statistically significant synergy with ZAP (P<0.05).

In the second approach we took, we systematically tested all the ISGs that reduced infection to less than 85% either in the control (11 genes) or ZAP (80 genes) cells and/or synergized with ZAP to a significant extent (Table 1; 69 genes with P<0.05), which resulted in a list of 84 unique ISGs. In addition, we tested viperin, ISG15 and ISG20, which have been shown to inhibit SINV [6], [29]–[32] but were not present or were non-inhibitory in our initial screen. For each ISG chosen, we prepared newly packaged VSV-G pseudotyped lentiviral particles, transduced triplicate wells of control and ZAP cells, and infected them with GFP-expressing SINV in three independent experiments. Data from each of the three screens are shown in Supplementary Table S3. We noted some variability in the percentage of infected cells in the three screens. Of the control cells expressing Fluc, 84.4 to 95.7% were infected, while 55.2 to 94.7% of the ZAP cells expressing Fluc were infected. In the three screens the reduction in the percentage of cells infected by ZAP expression alone ranged from −4.6 to 29.2% (compared to 8.4 in the primary screen and 27.1 in the confirmatory screen of the 23 ISGs). We utilized the same ANOVA model to identify synergistic partners of ZAP with a significant P value of <0.05 (see Methods and Supplementary Table S3). In the three experiments, 11 (C5orf39, MDA5, IRF1, IRF2, IRF7, LAMP3, MYD88, PRIC285, PSMB9, SAMD4A and SSBP3), 18 (C5orf39, CCDC109B, RIG-I, DEFB1, GCH1, IFI44L, MDA5, IL28RA, IRF1, IRF2, IRF7, ISG15, LMO2, MAP3K14, MKX, PCTK2, PMAIP1 and VAMP5), and 11 (C5orf39, RIG-I, MDA5, IL28RA, IRF1, IRF2, IRF7, MAP3K14, MYD88, PSMB9 and SSBP3) ISGs demonstrated a greater effect against SINV in combination with ZAP than ZAP or ISG expression alone (Supplementary Table S3). Among the synergistic hits, 11 genes (C5orf39, 4336 Via Linda Del Sur, Encinitas, CA 92024IL28RA, IRF1, IRF2, IRF7, MAP3K14, MDA5, MYD88, PSMB9, RIG-I and SSBP3) showed up in two or more of the replicate larger confirmatory screens (Table 3). Of the genes identified as synergistic in the confirmatory screen of the 23 ISGs nine (C5orf39, IRF2, IRF7, MDA5, MYD88, PSMB9, RIG-I, SAMD4A and SSBP3) were found to significantly up-regulate the antiviral function of ZAP in at least one of the larger confirmatory screens (Table 3).

**Table 3:** Summary of ISGs that showed significant synergy with ZAP in the top-23 screen and the larger confirmatory screens.

Gene Symbol	Top 23 Screen	Larger Screen #1	Larger Screen #2	Larger Screen #3	Pooled Larger Screens
BATF2	NT^1^				✓^2^
BTN3A3	✓				
C10orf10	NT				✓
C4orf33	NT				✓
C5orf39	✓	✓	✓	✓	✓
CCDC109B	NT		✓		✓
CCDC75	NT				✓
CTCFL	NT				✓
CXCL9	✓				
DDIT4	NT				✓
DEFB1	NT		✓		
FAM70A	NT				✓
GBP5	NT				✓
GCH1	NT		✓		
IFI44L	NT		✓		
IFIT5	✓				
IFITM3	✓				
IL28RA			✓	✓	✓
IRF1	NT	✓	✓	✓	✓
IRF2	✓	✓	✓	✓	✓
IRF7	✓	✓	✓	✓	✓
ISG15	NT		✓		✓
LAMP3	NT	✓			
LMO2	NT		✓		
MAP3K14	NT		✓	✓	✓
MDA5	✓	✓	✓	✓	✓
MKX	NT		✓		
MYD88	✓	✓		✓	✓
PCTK2	NT		✓		
PHF11	✓				
PMAIP1	NT		✓		
PRIC285	NT	✓			
PSMB9	✓	✓		✓	
RIG-I	✓		✓	✓	✓
SAMD4A	✓	✓			
SMAD3	✓				
SSBP3	✓	✓		✓	
UBE2L6	NT				✓
UPP2	✓				
VAMP5	NT		✓		✓

1 NT refers to a gene that was not tested in the particular screen.

2 A tick mark represents a gene that significantly synergized with ZAP (P<0.05).

To combine the data from the three larger confirmatory screens of 87 ISGs, we normalized the data within each of the larger screens and performed a statistical analysis for synergy on the combined data, adjusting the P values for multiple comparisons (Benjamini & Hochberg's adjustment). Among the 87 ISGs tested in three individual experiments, 21 (IRF2, C5orf39, RIG-I, DDIT4, ISG15, IL28RA, MAP3K14, IRF7, BATF2, CCDC109B, MDA5, UBE2L6, IRF1, CTCFL, C10orf10, MYD88, CCDC75, GBP5, C4orf33, VAMP5, FAM70A) were found to positively regulate ZAP activity against SINV (Supplementary Table S3). Combining the results of the screen of 23 ISGs with the combined analysis of the larger confirmatory screens reveals that a total of 31 ISGs (BATF2, BTN3A3, C10orf10, C4orf33, C5orf39, CCDC109B, CCDC75, CTCFL, CXCL9, DDIT4, FAM70A, GBP5, IFIT5, IFITM3, IL28RA, IRF1, IRF2, IRF7, ISG15, MAP3K14, MDA5, MYD88, PHF11, PSMB9, RIG-I, SAMD4A, SMAD3, SSBP3, UBE2L6, UPP2, VAMP5) demonstrated synergistic antiviral activity with ZAP.

### Knockdown of basal ZAP and IRF2 levels enhances SINV infection

**Figure 5:**
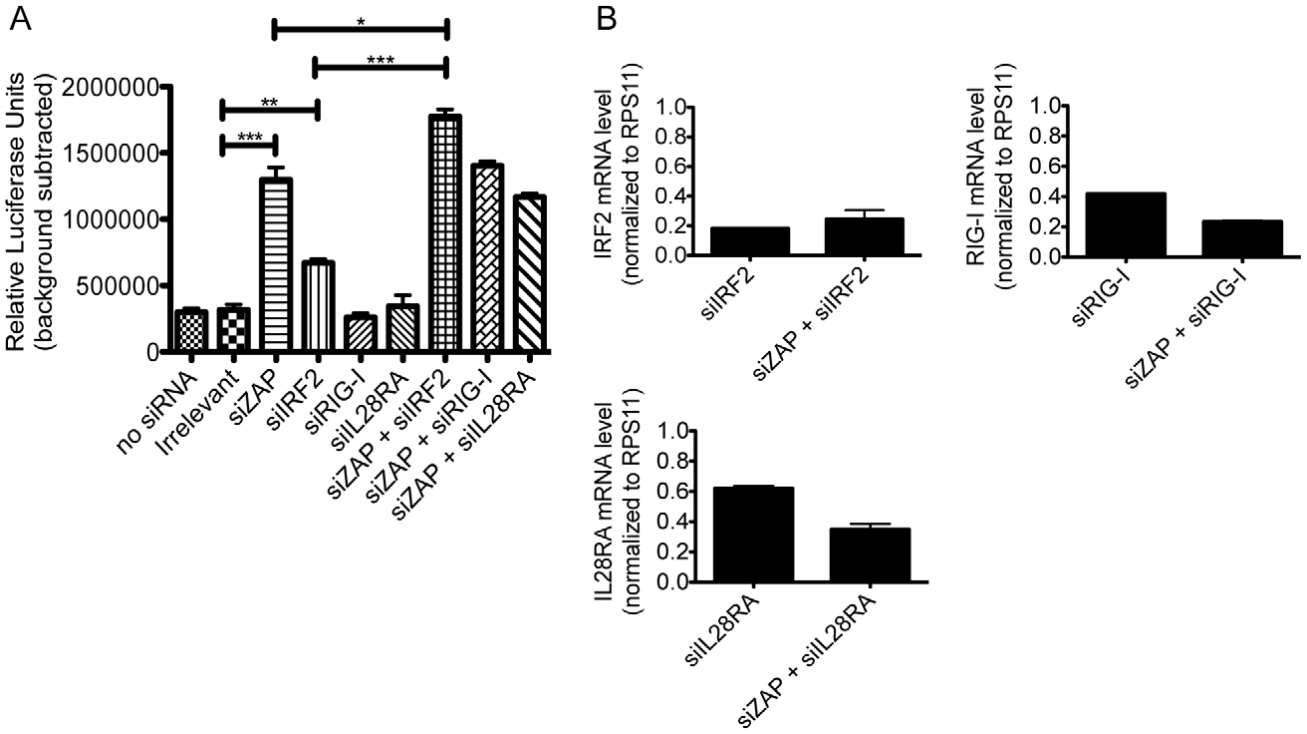
Validation of synergy between ZAP and the top three ISGs in a knockdown system. A) Triplicate wells of Huh-7 cells were transfected with irrelevant siRNA, ZAP-specific siRNA, ISG-specific siRNA that targets IRF2, RIG-I or IL28RA, or siRNAs that target both ZAP and an ISG. ISG-specific siRNA was added to cells again on the second day after seeding. Forty-eight h after initial siRNA transfection, cells were infected with Toto1101/Luc (moi = 5). Viral replication was determined by firefly luciferase activity 4 h after infection. Huh-7 cells that were not transfected with siRNA were included as a negative control. Means and standard deviations of triplicate samples are shown. Asterisks indicate mean values statistically different between two siRNA treatments (unpaired t test, *, P<0.05; **, P<0.01; ***, P<0.001). B) Forty-eight h after initial siRNA transfection, total RNA was extracted from the cells and used to generate cDNA. RNA levels of IRF2, RIG-I, IL28RA and RPS11 were measured by real-time PCR. The ISG mRNA levels were normalized with that of RPS11, and the ISG mRNA levels in irrelevant siRNA-transfected cells were set as 1. Data are means +/− SD of one experiment in triplicate.

Among the genes that synergized with ZAP, IRF2, RIG-I (also known as DDX58) and IL28RA demonstrated the greatest difference in infection level in the control compared to ZAP cells and consistently showed up in the secondary screens, suggesting an important role for these ISGs in modulating ZAP function. To validate their synergistic interactions with ZAP, we transiently knocked down IRF2, RIG-I and IL28RA with siRNA in the presence or absence of ZAP silencing in Huh-7 cells, which were then infected with SINV expressing a luciferase reporter. Comparing untransfected cells to irrelevant siRNA-treated cells, we found that siRNA transfection had no general effect on the level of infection determined by luciferase assay (Fig. 5A). Silencing of ZAP rescued SINV replication significantly (Fig. 5A) which was shown previously in 293T cells [13]. Knockdown efficiency was measured by qRT-PCR; ∼80% silencing of the long isoform of ZAP and ∼30–70% silencing of the short isoform were achieved. Silencing of RIG-I and IL28RA had no significant effect on infection compared to irrelevant siRNA transfection, suggesting that basal expression of these ISGs does not inhibit SINV (Fig. 5A). IRF2 silencing enhanced viral replication although the effect was not statistically significant in another similar experiment using a higher moi (Fig. 5A, data not shown). ISG knockdown efficiency was confirmed by qRT-PCR and ranged from 40 to 80% (Fig. 5B). In addition, cell viability was similar among siRNA-transfected cells, indicating that the difference in infection levels between samples was not due to siRNA-induced cytotoxicity (data not shown). When both ZAP and IRF2 were knocked down, viral replication was significantly increased compared to ZAP or IRF2 silencing alone, which supports the results obtained in the ISG overexpression screen and suggests that endogenous ZAP and IRF2 might interact in a synergistic manner (Fig. 5).

## Discussion

We previously determined that ZAP could synergize with one or more factors induced upon treatment of cells with IFN-α to mediate potent antiviral activity against the prototype *Alphavirus*, SINV [22]. In the present study, we screened a lentiviral library expressing over 350 individual ISGs [28] to identify those factors capable of synergizing with ZAP to confer an antiviral state within the cell. For the screen we chose BHK-21 cells, known to be defective in the IFN pathway [23]–[27], to minimize any effects due to IFN production within the cell culture system. We screened the panel of ISGs for their antiviral activity against SINV in two related BHK-21 derivatives, one transduced with the parental retroviral vector expressing the zeocin resistance gene (control cells) and one transduced with the retroviral vector expressing the amino terminal zinc finger-containing domain of rat ZAP fused to the zeocin resistance gene (NZAP-Zeo, ZAP cells).

Of the more than 300 ISGs with sufficient cell numbers and transduction efficiency for analysis, a small number had clear antiviral activity in the control cells in the absence of ZAP overexpression; 11 factors mediated inhibition resulting in an infection rate lower than an arbitrary cutoff of 85% (Fig. 1). Of all the ISGs, IRF1 demonstrated the most robust inhibition (infection percentage >11 SD below the mean in the control cells), and did so independently of ZAP overexpression; in both cell types less than 5% of the cells became infected. However, IRF1 did not restrict SINV replication to similar levels in the absence of ZAP overexpression in the confirmatory screens of 87 genes. In general, the 87 ISG confirmatory screens had lower transduction efficiencies, likely due to the use of a different transfection reagent in order to prepare the lentiviral particles, and it is possible that ISG expression levels are affected by the number of lentiviral particles entering each cell. The IRF1 transcription factor was previously found to exhibit antiviral activity against a number of viruses, including the alphaviruses Venezuelan equine encephalitis and chikungunya viruses [28], [33]. A component of the IFN-λ receptor (IL28RA) was the next most potent antiviral ISG in the control cells, resulting in an infection rate of less than 60% (>5 SD below the mean). This suggests that the IFN-λs may play an important role in defense against alphaviruses, and their role in preventing disease due to alphavirus infection deserves further study.

In contrast to the results in the control, a large number of ISGs exhibited antiviral activity in the ZAP cells, with 80 ISGs, more than a quarter of those analyzed, meeting the arbitrary cutoff of 85% infected (Fig. 1). The ability of many ISGs to function more effectively in the presence of ZAP (see also Fig. 2) suggests that in addition to possible direct interactions with the individual ISGs to mediate antiviral activity, ZAP may function to alter the intracellular milieu in some manner, rendering it more permissive for ISG function. It is of interest that ZAP expression is induced directly upon IRF3 activation [34], prior to the ISG upregulation that occurs in response to IFN-β. This early expression of ZAP might alter the environment and prime the cell for more potent ISG activity. In addition, it is also of interest that the short isoform of ZAP was recently found to interact with the cytosolic PAMP sensor, RIG-I (also known as DDX58) and to enhance IFN-β production upon RIG-I engagement [35]. While ZAP may be facilitating IFN production in these cells, which have an as yet undefined defect(s) in IFN production, it is unlikely that any increased IFN production is sufficient to induce a potent antiviral state, since the expression of ZAP in the control cells resulted in only a small to modest reduction in permissiveness to SINV infection. Further studies are required to determine the direct and global mechanisms by which ZAP is able to enhance ISG antiviral function.

For the 292 genes with data for analysis in both the control and ZAP cells we found that 69 ISGs showed statistically significant synergy in combination with ZAP (Table 1). Interestingly, we found no evidence for antiviral synergy with ISG20, which previously was shown to have greater than additive antiviral activity with ZAP [6]. Moreover, viperin did not demonstrate any antiviral activity against SINV in our confirmatory screens, whereas ISG15, although not antiviral by itself, demonstrated synergistic antiviral activity with ZAP in one of our confirmatory screens of the 87 genes. Possible explanations for these discrepancies with previous reports [6], [29]–[32] are the approaches utilized (overexpression versus gene silencing) and the cell types used for assessment of antiviral activity. Since we overexpressed the human ISG library in a hamster cell line, some of the ISGs might not be active or synergistic with ZAP due to the lack of compatible co-factors. Human ISG15 might not function well with its conjugating enzyme UbE1L of the hamster species, which has been demonstrated to play an important role in modulating ISG15 activity against SINV [30]. In addition, the overexpression system allowed us to identify factors that blocked infection primarily at early steps such as entry, translation and RNA replication. Due to the high moi we used in the screens, almost all the cells were infected in the first round and therefore could not be infected again by newly synthesized virus, which could mask any effects on virus production, release or spread. A previous study has shown that viperin inhibited SINV production, which could explain why it was not identified in our screens [29].

Since our primary screen was performed in singlicate wells, we wanted to confirm the synergy for the top ranking “hits”. We ranked the ISGs based on the magnitude of the difference in the percentage of infected cells expressing the ISG compared to the percentage of infected cells expressing both ISG and ZAP (Fig. 3). The top 23 were chosen for confirmatory testing (Fig. 4). Here, using 2-way ANOVA analysis to assess for synergistic activity, we found that 16 ISGs demonstrated a statistically significant synergistic effect with ZAP against SINV (Table 2). Interestingly, of the 23 ISGs chosen for validation, the majority were unable to reduce the percentage of infected cells to lower than 85% in the control cells, while all did so in the presence of ZAP. Given that we chose the ISGs for follow up based on the magnitude of the difference between cell types, this is not surprising. However, as can be seen in Figs. 1 and 2, the majority of ISGs that demonstrated anti-SINV activity in the ZAP cells were unable to reduce infection percentages to below 85% in the control cells. Whether this holds true in additional cell types will be important to determine.

In order to identify additional ISG candidates that synergize with ZAP, all the genes that exhibited inhibitory effects against SINV in the presence or absence of ZAP, and those identified as potentially synergistic in the primary screen were tested in a larger screen of 87 genes in three independent experiments. Variability was observed between the experiments, as the list of identified synergistic ISGs was not completely agreeable between the experiments. It is likely that the ISGs work in complex with other factors, and as such their individual expression with ZAP might not have dramatic effects on SINV replication. As a result, their effects might be easily affected by variables such as transduction efficiency of lentiviral particles, cell confluency and the ratio of SINV particles to lentiviral integrations per cell. Although the results were not identical between the experiments, there were 5, 8 and 8 genes in common for screens 1 and 2, screens 2 and 3, and screens 1 and 3, respectively (Table 3). The overlap of identified ISGs was statistically significant based on Fisher's exact tests (p = 0.045, 3.17×10^−7^ and 8.6×10^−5^), which indicates that these genes synergized with ZAP consistently and are important candidates for mechanistic follow up.

To demonstrate ZAP-ISG synergy using an alternative approach, we investigated the effects of double gene knockdown on SINV. Among the three ISGs tested, IRF2 and ZAP knockdown significantly enhanced viral replication compared to ZAP silencing alone, suggesting that IRF2 might positively regulate ZAP function. However, we did not observe a significant increase in viral replication upon silencing RIG-I or IL28RA in combination with ZAP, which might due to a number of factors. First, the antiviral activity of endogenous ZAP was large, potentially masking any additional effect caused by ISG silencing. Similar results were observed in 293T and Huh-7.5 cells (data not shown). Second, basal expression of ISGs might be low, limiting their impact on SINV replication and therefore precluding detection of synergy. Overexpression of the ISGs in an inducible cell line might provide a better system for validation of ZAP-ISG synergy as previously reported [6]. Alternatively, synergy could be examined in the context of IFN treatment, where expression of the ISGs would likely be increased compared to basal levels. However, treatment with type I IFN would induce expression of many ISGs followed by global changes in the antiviral state of the cells. As a result, it could be difficult to study the effects of a specific ISG. Finally, knockdown of RIG-I and IL28RA in Huh-7 cells was not as efficient as knockdown of IRF2, which might explain why silencing of the former ISGs did not affect SINV replication.

The mechanism by which ZAP might synergize with each of these ISGs is uncertain and requires further experimentation. ZAP and a synergistic ISG could target the same virus life cycle step or could target different viral steps to result in synergistic inhibition. Interestingly, some of the ISGs we identified as synergistic with ZAP are known components or regulators of RNA sensing and IFN induction pathways (IRF7, MYD88, MDA5, RIG-I, IRF2). Thus in addition to its interaction with and enhancement of RIG-I-mediated signaling and subsequent IFN-β production [35] one might speculate that ZAP broadly targets components of the innate immune PAMP recognition pathways to facilitate the establishment of the cellular antiviral state. Alternatively, it is likely that some ISGs enhance the antiviral activity of ZAP indirectly through their ability to induce expression of other ISGs, which then synergize with ZAP. It would be interesting to determine whether de novo synthesis of transcripts is required for ISG-ZAP interactions. Additional biochemical, cell biological, gene expression and signaling pathway analyses will be required to address whether the ISG-ZAP synergistic activity is due to direct or indirect interaction, and whether the synergistic ISGs and ZAP target distinct SINV life cycle steps. Future studies addressing whether these ISGs and ZAP can mediate synergistic antiviral activity against other *Alphavirus* genus members will be crucial for considering potential novel prevention or treatment strategies for these important pathogens.

## Materials and Methods

### Cell lines

BHK-21 derivatives constitutively expressing the zeocin resistance gene (BHK/HA-Zeo), or the amino terminal 254 amino acids of the rat ZAP protein fused to the zeocin resistance gene (BHK/NZAP-Zeo) were previously described [22] and were maintained in minimal essential medium (MEM) containing 7.5% fetal bovine serum (FBS) and 200 µg/ml zeocin. The BHK-21 cell line utilized for SINV titrations [3] and 293T cells utilized for lentiviral pseudoparticle production [36] were maintained as previously described. The Huh-7 cell line utilized for siRNA transfections were maintained in Dulbecco's Modified Eagle Medium (DMEM) containing 10% FBS and 1× non-essential amino acids (NEAA).

### Virus stocks and infections

SINV expressing EGFP from a duplicated viral subgenomic promoter (SINV TE/5′2J-GFP) [37] and SINV expressing firefly luciferase as a fusion with nsP3 (Toto1101/Luc) [3], were generated by electroporation of BHK-21 cells with in vitro-transcribed RNA and was titered on BHK-21 cells as previously described [3]. The moi of infection was calculated based on BHK-21-derived titers. Infections were conducted at 37°C for 1 h (with intermittent rocking) in a minimum volume of Dulbecco's phosphate buffered saline (DPBS) containing 1% FBS. All work with SINV was carried out under BSL2 conditions; incubation with vesphene or bleach was utilized for virion inactivation.

### The ISG library and preparation of VSV-G pseudotyped lentiviral stocks

A library containing over 350 ISG cDNA clones inserted in a lentiviral backbone was previously described [28]. Briefly, in this TRIP-based [38] lentiviral expression library, the CMV promoter drives expression of a transcript encoding the ISG, followed by an IRES and sequences encoding the TagRFP protein. Lentiviral particles pseudotyped with VSV-G were prepared by FuGENE- or X-tremeGENE 9- (Roche) mediated cotransfection of 293T cells with the pTRIP.CMV.IVSb.ISG.ires.TagRFP proviral plasmid, HIV-1 *gag-pol* and VSV-G DNAs as described [28]. After 6 h, the medium was replaced, and 48 h after the transfection, the medium was harvested and adjusted to contain 4 µg/ml polybrene and 20 mM HEPES, pH 7. After clarification by centrifugation single use aliquots were stored at −80°C. All lentivirus work was carried out under BSL2 conditions; incubation with vesphene or bleach was utilized for pseudoparticle inactivation.

### Transduction of BHK/HA-Zeo and BHK/NZAP-Zeo cells and SINV challenge

Cells were seeded in 24 well plates (5×10^4^ cells/well) one day prior to transduction. On the day of transduction, the medium was changed to one ml of MEM containing 3% FBS, 1× nonessential amino acids, 20 mM HEPES and 4 µg/ml polybrene. Pseudoparticles (100 µl) were added to the wells and the cells were transduced by spinoculation (1,500 g for 1 h, 37°C). After overnight incubation, the medium was changed to MEM containing 7.5% FBS and 200 µg/ml zeocin. Forty-eight h after transduction, the cells were infected with SINV TE/5′2J-GFP (moi = 5), using an inoculum of 100 µl per well. For each cell type, controls that were left untransduced, uninfected or both untransduced and uninfected were included for the purposes of setting the flow cytometry gates for RFP and GFP positivity. After 8 h of infection, the medium was removed and the cells were washed with DPBS, harvested in Accumax Cell Aggregate Dissociation Medium (eBioscience), and collected by centrifugation in 96-well format as described [28]. The cell pellets were resuspended in 100 µl DBPS containing 1% FBS, to which an equal volume of 4% paraformaldehyde in PBS was added. After fixation at 4°C for at least 30 min, the cells were collected by centrifugation and resuspended in 200 µl DPBS containing 3% FBS for storage at 4°C in the dark until flow cytometric analysis.

### Flow cytometry

A BD-LSRII equipped with 488 (GFP) and 561 (TagRFP) nm lasers and a High Throughput sampler (BD Biosciences) was utilized for flow cytometric data acquisition. Analysis was carried out using FlowJo software (Treestar). After gating on live, singlet cells based on forward and side scatter, a four quadrant plot was generated using the untransduced, uninfected cells such that ≥98% of the cells fell in quadrant 4 of the RFP vs. GFP plot (GFP and RFP negative). Compensation, defined by untransduced, uninfected (negative), untransduced, infected (GFP+ only), and transduced, uninfected (RFP+ only), was applied to all the samples. The compensated untransduced, uninfected sample was then utilized to generate a four-quadrant plot as above and this was applied to all samples. The cell counts from these quad plots were utilized for the Poisson regression analysis of the primary screen data (Table 1). To determine the percentage of cells that were transduced (RFP+), and amongst those, the percentage infected (GFP+), the live, singlet, compensated negative control sample was viewed as histograms to set the GFP+ and RFP+ gates for all the samples. The % infected for each sample, defined as the percentage of GFP+ cells within the RFP+ population, was obtained by applying the GFP+ gate to the RFP+ gate in all samples. This value was utilized for Figs. 1–4 and for the ANOVA regression analysis of the 23 ISGs with the greatest difference in % infection between control and ZAP cells and the 87 synergistic and/or antiviral ISGs that underwent confirmatory testing (Table 2 and Supplementary Table S3).

### Statistical analyses

For the purposes of our analysis we considered two variables producing an effect greater than the sum of their individual effects as exhibiting synergy. Both the primary screen data and the data obtained in the confirmatory testing of the 23 and later 87 ISGs were subjected to statistical analyses.

#### Poisson regression on the primary screen data

For the primary screen data, the fact that our data set only consisted of a single value for the percentage of infected cells for each ISG in the control and ZAP cells precluded two-way ANOVA analysis. However, count data were available from the 4 quadrant flow cytometry data, where quadrant 2 (Q2) was RFP positive (transduced, ISG-expressing) and GFP positive (infected) while quadrant 1 (Q1) was RFP positive (transduced), but GFP negative (uninfected). In our data, the rate was defined as the count of RFP+ cells that were infected (GFP+), divided by the number of cells exposed (total number of RFP+ cells). Using the quadrant count data, Q2/(Q1+Q2) represents the rate of infection for each sample, and Poisson regression was utilized, treating the number of exposed cells as an offset, as shown in equation (1):
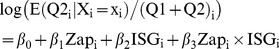
(1)In this equation, Q2_i_ and (Q1+Q2)_i_ were as described above. E(Q2_i_|X_i_ = x_i_) represents the expected number of RFP+ cells that were infected (GFP+) given the values of independent variables, including intercept, main effects of ZAP, ISG and their intersection term. If the coefficient (β_3_) of the interaction term of ZAP and ISG is negative and significant, it implies that the relative ratio of infection in the ZAP+ISG group is statistically significantly less than the product of the relative ratios in the ZAP only group and that in the ISG only group. The Benjamini–Hochberg procedure was used to adjust P values for multiple comparisons.

#### Two-way ANOVA for the confirmatory testing

For the confirmatory testing of the 23 ISGs with the greatest difference in % infection between control and ZAP cells, in which there are three replicates for each group, we utilized 2-way ANOVA to model the percentage of transduced (RFP+) cells that were infected (GFP+). The covariates include intercept, main effects of ZAP, ISG and their intersection term. Those ISGs having a negative estimated coefficient on the interaction term and a post-hoc adjusted (Hommel's adjustment) P value less than 0.05 were considered to significantly synergize with ZAP.

For the confirmatory testing of the 87 synergistic and/or antiviral ISGs identified by Poisson regression, in which there are three replicates for each group and three screens (we conducted the same experiment three times) for each gene, we utilized 2-way ANOVA to model the percentage of transduced (RFP+) cells that were infected (GFP+) separately for each screen. The covariates include intercept, main effects of ZAP, ISG and their intersection term. Those ISGs having a negative estimated coefficient on the interaction term and a p-value less than 0.05 were considered to potentially synergize with ZAP.

To combine these three screens (resulting in nine replicates for each condition and greater statistical power), we firstly normalized the percentage of transduced cells that were infected within each screen (Z-score normalization). The values were further normalized between screens such that the obtained scores would have a mean of zero and a variance of 1 so that measures from different screens on the same gene would be comparable. Finally, we utilized 2-way ANOVA to model the resulting score. The covariates include intercept, main effects of ZAP, ISG and their intersection term. Those ISGs having a negative estimated coefficient on the interaction term and an adjusted (Benjamini-Hochberg adjustment) p-value less than 0.05 were considered to significantly synergize with ZAP.

#### Software

All statistical analyses for synergy were carried out using R language version 2.12 (www.r-project.org). Unpaired two-tailed *t*-tests were performed in the PRISM software (GraphPad).

### siRNA transfection

For small interfering RNA (siRNA) transfection, Huh-7 cells were seeded the day before at 5×10^4^/well in a 24-well plate. Triplicate samples were transfected using Lipofectamine RNAiMAX transfection reagent (Invitrogen) according to the manufacturer's protocol, with either irrelevant siRNA, 6 pmol of ZAP siRNA, 30 pmol of IRF2/RIG-I/IL28RA siRNA, or 6 pmol of ZAP siRNA and 30 pmol of IRF2/RIG-I/IL28RA siRNA per well. The total amount of siRNA used per well was kept constant (36 pmol) by addition of irrelevant siRNA. One day post-transfection, cells were again treated with 30 pmol of IRF2/RIG-I/IL28RA siRNA to achieve efficient knockdown. siRNA duplexes were ZAP specific [13], IRF2 specific (ON-TARGETplus SMARTpool siRNAs L-011705-00; Dharmacon), RIG-I specific (ON-TARGETplus SMARTpool siRNAs L-012511-00; Dharmacon), IL28RA specific (ON-TARGETplus SMARTpool siRNAs L-007981-00; Dharmacon), or irrelevant (ON-TARGETplus Non-targetting pool D-001810-10; Dharmacon). Forty-eight hours after the first siRNA transfection, the cells were infected with Toto1101/Luc (moi = 5), using an inoculum of 100 µl per well. After 4 h of infection, the medium was removed and the cells were lysed for luciferase assay as previously reported [22]. Cell viability at the time of infection was determined using the CellTiter-Glo (Promega) assay according to the manufacturer's recommendations.

### Quantitative Reverse-Transcription Polymerase Chain Reaction (RT-PCR)

RNA was prepared using the RNeasy mini kit (Qiagen) and quantified by absorption spectrophotometry using a NanoDrop. RNA (1 µg) was used as a template for reverse transcription using SuperScript III (Invitrogen) and random hexamers. Five µL of 10-fold-diluted cDNA was used in a SYBR Green qPCR assay (Roche) on the LightCycler 480 Real-Time PCR System (Roche). The primers specific for RPS11, IRF2 and RIG-I were as described [39], Primers for ZAP long and short isoforms were from Qiagen (QuantiTect Primer Assay Hs_ZC3HAV1_1_SG and Hs_ZC3HAV1_vb.1_SG), while IL28RA primers were forward primer 5′-AAGACCCTATTTCCAGTCACTCC-3′ and reverse primer 5′- GAACGTGTAGATGGTTCTGGC-3′. Expression was normalized to that of the housekeeping gene RPS11 and to expression in cells treated with irrelevant siRNA.

## Supporting Information

Table S1 **Contains the data from the primary screen and the Poisson regression analysis.** The contents of each Tab are as follows: **Tab 1:** For each cell type (Control or ZAP) the percentage of the infected, ISG-expressing cells (%GFP-positive in RFP-positive population) is shown for each ISG. Samples with less than 5000 cells for analysis, or <30% transduction efficiency are excluded. A normalized value for each ISG within the cell type, defined by the mean and standard deviation of all the samples, was calculated in excel by the STANDARDIZE function. **Tab 2:** The 292 ISGs for which data were available in both the Control and ZAP cells were sorted based on the percentage of infected cells in the Control cells. The difference in the percentage infected (% infected in the control cells minus % infected in the ZAP cells) is also displayed. **Tab 3:** The data in Tab 2 is sorted by the magnitude of the difference in percentage of cells infected in the two cell types. **Tab 4:** For each ISG, the number of cells in quadrant 1 (RFP+, GFP−) and quadrant 2 (RFP+, GFP+) are shown for the two cell types. The estimated interaction coefficient and corresponding P value (and adjusted P value) obtained in the Poisson regression analysis are shown for each ISG. **Tab 5:** The results from the Poisson regression analysis (from Tab 4) on the 292 ISGs are displayed, sorted by the magnitude of the estimated interaction coefficient.
(XLS)

Table S2 **Contains the data from the confirmatory testing of 23 ISGs and the ANOVA analysis.** The contents of each Tab are as follows: **Tab 1:** For each cell type (Control or ZAP) the percentage of infected, ISG-expressing cells (%GFP-positive in RFP-positive population) is shown for each of the 23 ISGs and Fluc control. The difference in the mean percentage infected is also shown. **Tab 2:** The difference in the mean percentage of cells infected in the two cell types (% infected in the control cells minus % infected in the ZAP cells) is shown for the 23 ISGs and Fluc control, sorted by the magnitude of the difference. **Tab 3:** The estimated interaction coefficients from ANOVA for the 23 ISGs and the corresponding P values are shown.
(XLS)

Table S3 **Contains the data from the confirmatory testing of 87 ISGs and the ANOVA analysis.** The contents of each Tab are as follows: **Tab 1:** This tab shows the results from the first of the three confirmatory screens of 87 ISGs. For each cell type (Control or ZAP) the percentage of infected, ISG-expressing cells (%GFP-positive in RFP-positive population) is shown. The data displayed includes the average %infected from each replicate, the standard deviation (SD), as well as the average transduction percentage and standard deviation (SD). The average number of cells analyzed after gating on singlet cells is also shown with the standard deviation (SD). The results of the ANOVA analysis are displayed at the bottom of the table. The estimate column refers to the magnitudes of the synergy effects. Those ISGs having a negative estimated coefficient on the interaction term and a P value<0.05 were considered to potentially synergize with ZAP. **Tab 2:** This tab shows the results from the second of the three confirmatory screens of 87 ISGs. **Tab 3:** This tab shows the results from the third of the three confirmatory screens of 87 ISGs. The 87 genes were divided into two groups for performing the transduction and infection steps of the screen. The genes belonging to each group are highlighted by different colors. **Tab 4:** This tab displays the ANOVA results from the combined normalized confirmatory screens of 87 ISGs. Those ISGs having a negative estimated coefficient on the interaction term and an adjusted P value<0.05 were considered to significantly synergize with ZAP.
(XLS)
